# Postpartum psychosis: a public involvement perspective across three continents

**DOI:** 10.1007/s00737-023-01347-8

**Published:** 2023-08-24

**Authors:** Jessica Mei Kay Yang, Kimneihat Vaiphei, Mercy Siliya, Thandiwe Mkandawire, Clare Dolman, Jessica Heron, Sally Wilson, Shivanand Yaresheemi, Danielle Kitney, Leah Bailey, Chloe Apsey, Olive Liwimbi, Robert Stewart, Harish Thippeswamy, Ian Jones, Genesis Chorwe-Sungani, Prabha Chandra, Arianna Di Florio

**Affiliations:** 1 Centre for Neuropsychiatric Genetics and Genomics, Cardiff University, Cardiff, UK; 2 Department of Psychiatric Social Work, National Institute of Mental Health and Neuro Sciences, Bengaluru, India; 3 Thyolo District Health Office, Thyolo, Malawi; 4 Mental Health Users and Carers Association, Blantyre, Malawi; 5 Section of Women’s Mental Health, Health Service and Population Research Department, Institute of Psychiatry, Psychology & Neuroscience, King’s College London, London, UK; 6 Bipolar UK, 32 Cubitt Street, London, UK; 7 Action on Postpartum Psychosis, Institute of Mental Health, University of Birmingham, Birmingham, UK; 8 Action on Postpartum Psychosis, PO Box 137, Swansea, UK; 9 National Centre for Mental Health, Cardiff University, Cardiff, UK; 10 Zomba Mental Hospital, Zomba, Malawi; 11 Division of Psychiatry, University of Edinburgh, Scotland, UK; 12 Kamuzu University of Health Sciences, Blantyre, Malawi

**Keywords:** Postpartum psychosis, Maternal mental health, Low- and lower-middle income countries, Public involvement, Cross-cultural

## Abstract

Postpartum psychosis is a psychiatric emergency that is currently not represented in diagnostic systems, to the detriment of people with lived experience. Engaging with stakeholders offers an important avenue to improve clinical practice and make research more impactful, by providing perspectives based on first-hand, expert experience. There is a paucity of reports on stakeholders’ engagement in psychiatry. Activities have thus far been limited to Western countries and there are few reports on postpartum psychosis. We report the results of public involvement activities (in the form of discussion groups) with key stakeholders in India, Malawi and the UK. These discussions centred around the clinical picture of postpartum psychosis and the terminologies used to describe these episodes. Seven major areas were highlighted: how postpartum psychosis is handled within services, common symptoms and characteristics, impact of episode, barriers to care, non-medical approaches, terminology and research areas of interest. According to the discussions, postpartum psychosis presents similarly across countries, although there are differences in access to services, approaches to mental health and terminologies used within and across countries. With this understanding comes the foundation for cross-cultural assessment, service improvement and a stakeholder-informed research agenda.

## Background

Postpartum psychosis is a severe postnatal mental illness, reported to affect around 1–2 for every 1000 childbirths worldwide (Perry et al. 2021), yet it is currently unrecognised by diagnostic systems. The term “postpartum psychosis” is typically used in the clinical context to describe a diverse collection of symptoms with an abrupt onset, traditionally within the first 2 weeks after childbirth. Common symptoms include mania, depression, mixed states and psychosis. In some cases, women will also experience neurological and motor difficulties (Yang et al. 2022).

The lack of an official recognition and standardised diagnosis has hindered research, in turn preventing improvements in early intervention and standards of care for women and their families (Di Florio et al. 2016). The lack of official status is particularly detrimental for low- and lower-middle income countries (LMICs). Here, the research into maternal mental health is even more limited than in high-income countries (WHO 2008), resulting in a strong bias in definitions, assessment criteria and tools towards the latter. For example, limited evidence suggests that the incidence of postpartum psychosis may be slightly higher in some LMICs such as Nigeria (2.5 in 1000) and India (2.6 in 1000) than the rates reported by studies conducted in high-income countries (VanderKruik et al. 2017).

It is becoming increasingly clear that engaging stakeholders at all stages is necessary to ensure high quality, relevant research (Minogue et al. 2018). Limited evidence suggests that service user-led research provides rich data in areas rarely researched in such detail. For example, women with lived experience of postpartum psychosis have contributed to crucial insight into why full recovery from an episode is often arduous (Heron et al. 2012).

Here we report on the use of discussion groups as a patient and public involvement (PPI) activity spanning three continents. PPI in this context is defined as a targeted consultation, whereby participants are contacted based on their affiliation with groups of interest and involvement is limited to specific activities (Doria et al. 2018). In contrast to research activities, discussion groups offer the opportunity for people with lived experience to engage in research project planning (Doria et al. 2018). The activities were conducted as part of a wider, ongoing collaboration of the International Postpartum Psychosis Consortium, consisting of academic professionals working in the area of postpartum psychosis across various continents. Given the considerable lack of reproducible research into postpartum psychosis, and in particular within LMICs, stakeholders were asked to input their perspective on the context in which postpartum psychosis occurs within each country, with the overall aim of enhancing research into postpartum psychosis across countries.

## Methods

### Design and stages of involvement

#### Stage 1: Initial discussions

All activities were based at three centres: National Institute of Mental Health And Neuro Sciences (NIMHANS; Bengaluru, India), Kamuzu University of Health Sciences (Blantyre, Malawi) and Cardiff University (Cardiff, Wales). The teams met once a month using video conferencing software for the duration of the project. Initial discussions focused on finalising the aims and questions for the discussion groups and anticipating any potential barriers to research. Questions were reviewed to ensure they were suitable across the three centres and would not trigger any negative emotional responses.

#### Stage 2: Discussion groups

For the PPI activities, stakeholders were split into three groups: people with lived experience of postpartum psychosis (PWLE), family and friends who supported someone during an episode of postpartum psychosis and healthcare professionals (HCPs) involved in the pathway to care. Each meeting consisted of 3–7 people and took place between June 2021 and July 2022. Discussions briefly comprised of an introduction on the aims of the discussion and providing oral (and/or written) consent to participate and for the discussion to be recorded so that transcripts could be made. Discussions then focused on ideas for future research, clinical manifestations and treatment course for postpartum psychosis and the terminology used to describe these episodes.

For this manuscript, two facilitators (JY and DK) identified relevant excerpts independently and organised them. These were then checked alongside two more facilitators (LB and CA) for agreement and were finalised with the wider team. Patient representatives (TM, CD, SW) facilitated and supervised the process, contributed to edits of the paper and are co-authors. The GRIPP2 short form checklist for the reporting of PPI activities was followed (Staniszewska et al. 2017).

### Participants

Number of participants within discussion groups for each centre are reported in Table 1.

**Table 1:** Total number of stakeholders and discussion groups

Stakeholder group	India	Malawi	UK
People with lived experience	38 (8)	10	10 (4)
Family and friends	20 (4)	7	4 (2)*
Healthcare professionals	6 (1)	5	11 (3)

Number in brackets denotes total number of discussion groups held for each group

*One individual from the family and friends group participated in a discussion with people with lived experience due to availability

#### India

People who obtained care for postpartum psychosis at NIMHANS perinatal psychiatry services and their family members were invited by phone. The consultation meetings were held at the weekly perinatal psychiatry services clinic, NIMHANS. In addition, we held a meeting with healthcare experts from health facilities that provide care to postpartum parents. The main language used in the meetings was Kannada. The family group included partners, mothers and mothers-in-law.

#### Malawi

Meetings with PWLE and their family members were held in Blantyre in Southern Malawi. In addition, we held a meeting with HCPs from health facilities within Blantyre that work with postpartum patients. At the start of the meetings, the purpose of the consultations was explained and expectations regarding respectful interactions and confidentiality were set out. Group meetings were held in English for HCPs and Chichewa for PWLE and family. The family group comprised of parents, partners and brothers.

#### UK

PWLE and family and friends were recruited through ongoing collaborations with local patient support organisation Action on Postpartum Psychosis and Health and Care Research Wales via social media. Group meetings were held online using video conferencing software in English. HCPs included health visitors, a perinatal pharmacist, perinatal psychologist, perinatal nurse, perinatal occupational therapist and a student midwife. The family group included only partners.

## Results

### Stage 1: Initial discussions

Two main barriers to cross-cultural research into postpartum psychosis were identified. First, literary and language barriers may impact definitions used and on reporting of symptoms. Second, due to significant differences in the pathway to care, it is difficult to draw comparisons across cultures.

It was therefore decided that diagnoses would need to be described using culturally appropriate terminology. To avoid confusion and improve knowledge of the context in which future research may take place, stakeholders would be consulted on the typical course of illness, pathway to care and terminology they used to describe an episode of postpartum psychosis. Initial discussions also identified the need to hold separate groups with family and friends who supported someone during an episode of postpartum psychosis, as people may have poor recall around their episode.

### Stage 2: Discussion groups

Seven overarching themes relating to the cross-cultural conceptualisation of postpartum psychosis and research into the condition emerged from the PPI activities: how postpartum psychosis is handled within services, common symptoms and characteristics, impact of episode, barriers to care, non-medical approaches, terminologies and research areas of interest. We report the main discussion points around these themes within each country.

#### India

PWLE and their families reported that they may not recognise the term “postpartum psychosis”, and, even if informed, they might not remember the name and refer to the condition as “psychosis”. Postpartum psychosis is often referred to as “depression”. The following terms were also used to describe postpartum psychosis in Kannada: “Sanni ageti” (postpartum mental disturbances), “Devva hididide” (possessed by ghost), “Bananti kayile” (postpartum illness), “Devaru bandeti” (possessed by god). Many stakeholders could identify the symptoms of postpartum psychosis but could not name the diagnosis. They said they preferred to describe symptoms as opposed to using medical diagnoses. Symptoms included sleep disturbances, behavioural changes, changes in daily functioning, inability or neglecting care for their new-born baby, mood changes, fearfulness and irritability. Some also noted hallucinations and delusions as symptoms of postpartum psychosis.

HCPs reported that people with postpartum psychosis delay in seeking professional assistance. It emerged that traditional/spiritual healers are usually the first point of contact for help for people with perinatal mental illness, as some may believe that supernatural or spiritual forces are to blame for the episode. Traditional healing practices like chanting, branding, fasting or prayer are frequently used for treatment. Family seek help from nearby healthcare services and get referred to the tertiary care centre only when they do not see improvement following traditional healings and practices.

Stigma, the belief that postpartum psychosis is not a psychiatric illness that requires medical intervention, lack of knowledge and understanding about the symptoms of postpartum psychosis were reported as barriers to care.

Research priorities differed between HCPs and PWLE. HCPs indicated a need to conduct research on a broad range of topics from disease mechanisms to clinical and service improvements, including attention to mother-child bonding and the role of magico-religious beliefs. PWLE emphasised the need for research and service delivery, including educating the community and traditional/spiritual healers about postpartum psychosis and addressing domestic violence. Families also highlighted the need for educating the community and reduction of stigma.

#### Malawi

PWLE and their families agreed that the general term “mental illness” is frequently used to refer to postpartum psychosis. Similar to discussions in India, communication based on symptoms as opposed to medical diagnosis was preferred. Descriptions of an evil spirit or possession were reported as often being used instead of medical diagnoses for postpartum psychosis, (severe) postpartum depression and postpartum obsessive-compulsive disorder. HCPs also mentioned that they would use different names for postpartum psychosis. Differences were attributed to variation in literacy and lack of standardisation in diagnosing mental illness among HCPs. Symptoms identified as connected to childbirth and postpartum psychosis included sleep problems and a mother’s inability to care for their new-born. A history of postpartum psychosis was mentioned as a common risk factor. Although people with postpartum psychosis seek assistance more rapidly than those with other mental health problems like schizophrenia, HCPs reported limited access to services, with unclear pathways to care and lack of official treatment guidance in the country. There was consensus in the discussions on the importance of family and support in raising children and more generally during an episode of postpartum psychosis.

Traditional medicine or prayer emerged as frequently used treatments for postpartum psychosis. Further, it transpired that traditional/spiritual healers are usually the first point of contact for help for people with mental disorders. All groups noted that some, typically people in the general population, may hold the belief that supernatural or spiritual forces are to blame for an episode of postpartum psychosis. Discussions among PWLE, family and HCPs yielded information that stigma, the belief that postpartum psychosis is not a psychiatric event requiring medical intervention and lack of knowledge, understanding and discussion as barriers to care. Other factors included families and communities attempting to cope with the episode on their own without seeking medical intervention.

HCPs reported on the wide spectrum of knowledge and handling of episodes by HCPs, including around diagnosis. This could hinder effective care through misdiagnosis or inappropriate treatment and maintenance. The impact of the episode was discussed by HCPs only. Areas for future research identified were mostly clinically focussed, including treatment, maintenance and characteristics of postpartum psychosis.

#### UK

In contrast to India and Malawi, labels for postpartum psychosis centred around clinical diagnoses. PWLE and partners often talked about not being given any label during the episode. PWLE noted that this and the use of alternative or more general labels may be a result of stigma.

Disordered sleep, mania, depression, distorted reality, hallucinations/delusions and the presence of situational/environmental risk factors were often cited as key characteristics of postpartum psychosis. HCPs focussed on onset indicators such as changes in behaviour and risk of harm whilst PWLE focused on concentration and physical symptoms. Notably, PWLE sometimes reported having insight into their illness at the time, whilst HCPs tended to report a loss of insight. Partners reported different characteristics still, namely “strange” behaviour, dilation of the pupils and comorbidities such as infection.

Service provision for new mothers with severe mental illness was said to vary significantly across the country. Although medication was often associated with negative experiences and side effects by PWLE and partners, medical intervention and inpatient admission to a mother and baby unit was considered the ideal.

Key barriers to care included lack of knowledge among HCPs and fear of social services. The need of a strong alliance between stakeholders emerged from all discussions. PWLE and partners highlighted the need for themselves to be proactive in diagnosis and treatment and a general lack of support and communication from HCPs throughout. HCPs noted how family and the wider support network may have specific views on treatment and care which do not align with what is recommended.

The importance of the difference between symptom remission, which could take months, and full recovery, which could take years was stressed in all discussions. Long-term consequences on relationships, sense of self and fear of recurrences were reported. Volunteering with third sector charities was reported as a positive impact. The main interests for research in the UK centred around causes and risk factors, with translational clinical research seen as an important focus.

## Discussion

Research into postpartum psychosis, particularly within LMICs, has been impeded by an absence of appropriate assessment tools, consistent diagnostic guidelines and reproducible results. To date, the literature on stakeholder perspectives of postpartum psychosis has been limited. We conducted PPI activities in the form of discussion groups with stakeholders across three different countries and cultures to begin tackling these gaps. As a starting point of discussion, we aimed to explore how postpartum psychosis is conceptualised in different cultures and to ensure that the research agenda is relevant to stakeholders.

Prior to these PPI activities, the intended research course was to create a postpartum psychosis assessment tool that was cross-culturally useful and contextually sensitive. After, it became clear that this research aim would not be so straightforward. There were cultural differences and technology and language barriers to consider. It is likely that three separate assessment tools, validated within each country, will be necessary to create the most reliable and effective tool to identify cases of postpartum psychosis. Comparability between countries will require ongoing cross-cultural collaboration with stakeholders.

**Fig. 1:**
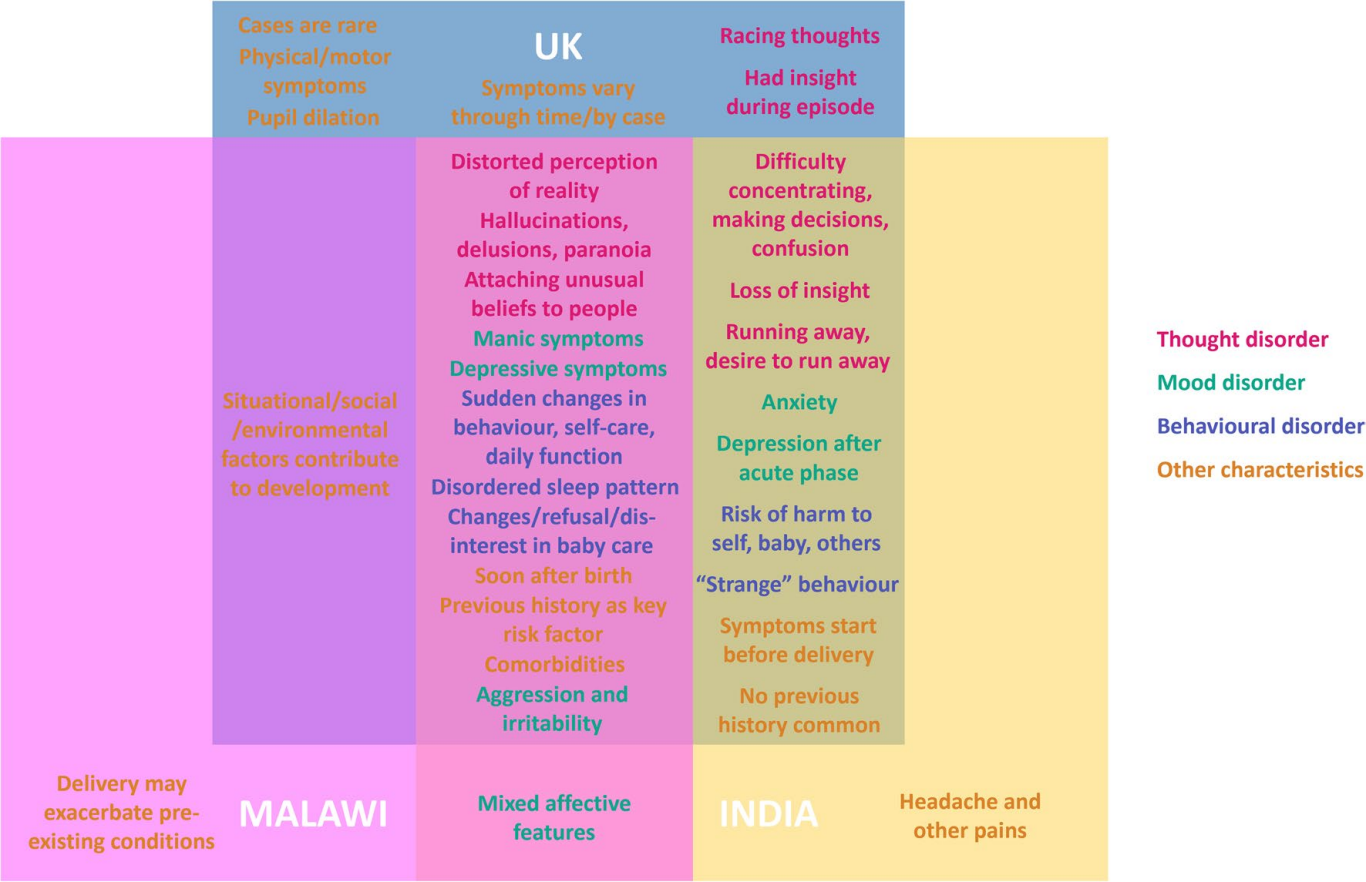
Venn diagram breakdown of common symptoms and characteristics of postpartum psychosis that were discussed by stakeholders in each country. Note: this is not an exhaustive list of symptoms and characteristics of postpartum psychosis that present within each country. It shows only the symptoms and characteristics that were reported within the constraints of the group discussion

Key outcomes included the knowledge that postpartum psychosis presents comparably across countries with similar symptoms and characteristics (Fig. 1), despite differences in labelling, conceptualisation, service provision and approaches to mental health. Given the differences in access to services, pathways to care and lack of official treatment guidance, the considerable similarities between countries were unexpected. Major differences in provision, access to services and approaches to care emerged not only between countries, but also within each country, making reproducible research particularly challenging. A previous report from Australia on stakeholder perspectives of postpartum psychosis supports many of our observations, including people having some insight at the time of the illness, the key role of sleep and stress and positive outcomes such as volunteering (Jefferies et al. 2021). Our observations in the UK regarding the negative experiences with medical treatment and difference between symptom remission and feeling recovered is also a reflection of previous literature (Heron et al. 2012).

India, Malawi and the UK were shown to share similar barriers to care and concerns about the impact of postpartum psychosis episodes. Earlier literature from India and the UK describes stigma and limitations of services as barriers to care (Banerjee et al. 2021; Heron et al. 2012). Similarly, in Australia, PWLE of postpartum psychosis report reluctance in reporting symptoms to HCPs (Jefferies et al. 2021). Differences in education and belief systems likely contribute to the differences in labels and treatments that were observed between groups and countries. The use of more general terminologies in place of medical diagnoses in India and Malawi has also been reported previously (Stewart et al. 2015; Thippeswamy et al. 2015).

The important role of traditional beliefs and healers in Malawi is consistent with previous literature which also reported a gradual cultural shift towards hospital-based care (Stewart et al. 2015), which may contribute to the observation that people with postpartum psychosis present to services more quickly than other conditions. Research in India has shown that beliefs in supernatural and spiritual causes are present but not predominant (Thippeswamy et al. 2015) which is interesting given that many will seek traditional healers for help first.

The need to include stakeholders in healthcare research has been highlighted consistently across all three countries (Heron et al. 2012; Minogue et al. 2018; Banerjee et al. 2021; Manda-Taylor et al. 2021). Having input from a broad range of stakeholders at this early stage was key, as views sometimes differed across groups. For example, discussions in India highlighted different research priorities between HCPs and PWLE and their families. Although there was agreement on the severity of the illness and accounts of symptoms, experiences and priorities differed between stakeholders. These discussions inform us of the direction that stakeholders believe research should take to improve standards of care whilst also demonstrating the significant contribution that stakeholders make to research.

To our knowledge, this project’s use of PPI activities to compare characteristics of a severe mental health condition across countries is the first of its kind. This work will influence future research by providing information on ascertainment methods, assessment and areas of research impact. It will also help account for cultural factors in research design, including biological studies. The cooperation and motivation of PWLE and families to engage was encouraging and this is perhaps attributable, in part, to the inclusion of family members. Familial relationships have been shown to be integral in the decision-making around research participation in maternal mental health within Malawi (Manda-Taylor et al. 2021) where families in particular are expected to provide support, as reported elsewhere (Stewart et al. 2015). Having active clinicians as part of the International Postpartum Psychosis Consortium was also imperative. For example, we would not have chosen to include family and friends without clinician input as the issue of poor recall would not have been highlighted.

There are some challenges to consider with this type of work. Attempting the same activity in different countries required extra care in considering language and cultural context. This meant understanding the typical language and context of stakeholders which consequently required discussion with those groups. Given that these discussions were held with small groups and at only one centre per country, it is possible that they were not representative of all experiences. This is especially apparent considering that most people had experience of Western-centric medical practices, either as a HCP or as a patient or relative.

## Conclusion

In conclusion, this article outlines a novel approach whereby PPI activities are used cross-culturally to take into consideration the views of different stakeholder groups and inform future research into postpartum psychosis. Even across cultures, postpartum psychosis presents with similar characteristics, despite cultural differences in services, labels and approaches to mental health. These discourses provide suggestions for ways research can improve standards of care and outcomes for people who experience postpartum psychosis, which is the ultimate goal.
